# Adoption of the Polish Bariatric and Metabolic Surgery Care Standards: a nationwide survey

**DOI:** 10.20452/wiitm.2024.17911

**Published:** 2024-11-15

**Authors:** Mateusz J. Świerz, Karolina Majdak, Andrzej Budzyński, Wiesław Tarnowski, Piotr Major, Małgorzata M. Bala

**Affiliations:** 2nd Department of General Surgery, Jagiellonian University Medical College, Kraków, Poland; Department of Hygiene and Dietetics, Chair of Epidemiology and Preventive Medicine, Jagiellonian University Medical College, Kraków, Poland; Systematic Reviews Unit, Jagiellonian University Medical College, Kraków, Poland; Department of Medical Sociology, Chair of Epidemiology and Preventive Medicine, Jagiellonian University Medical College, Kraków, Poland; Department of General and Oncological Surgery, Ludwik Rydygier Memorial Hospital, Kraków,, Poland; Centre of Postgraduate Medical Education, Orlowski Hospital, Warszawa, Poland

**Keywords:** bariatric surgery, metabolic surgery, obesity management, perioperative care, standards of care

## Abstract

**INTRODUCTION:**

Metabolic and bariatric surgery (MBS) is the most effective treatment for severe obesity, providing substantial weight loss and improvement in obesity-related comorbidities. In 2020, the MBS Chapter of the Association of Polish Surgeons issued the Bariatric and Metabolic Surgery Care Standards to guide patient management.

**AIM:**

The aim of the study was to asses implementation of the standards in Polish surgical departments and identify factors associated with better compliance.

**MATERIALS AND METHODS:**

An online survey was distributed between August and December 2022 to 46 Polish surgical departments performing MBS. The survey included 62 questions covering general center characteristics, and pre-, peri-, and postoperative care. Descriptive statistics summarized the center characteristics and linear regression models analyzed the factors influencing compliance.

**RESULTS:**

Thirty-six centers completed the survey, with a mean (SD) compliance score of 86.8% (9%) (29.5 of 34 criteria), and individual scores ranging from 70.6% to 100%. As many as 66.7% of the centers answered at least 80% of the questions. The areas with compliance below 80% included availability of adapted radiology facilities, constant availability of emergency radiology diagnostics, screening for metabolic markers, assessment of obstructive sleep apnea risk, obtaining patient declaration of abstaining from smoking, providing dates for at least 1 dietary consultation upon discharge, requiring at least 2 consultations with an experienced dietician, and employing a surgeon with bariatric certificate of excellence. Significant predictors of better compliance included the number of surgeons performing MBS and participation in the KOS-BAR program (a program of complex specialist care over patient undergoing bariatric surgery).

**CONCLUSIONS:**

The Bariatric and Metabolic Surgery Care Standards are moderately well adopted by Polish surgical departments, with improvements needed in specific areas. Unifying the standards may enhance patient outcomes.

## INTRODUCTION

Obesity is a global health problem affecting 16% of adults worldwide as of 2022, which amounts to over 890 million people.^1^ In Poland, the obesity rate is 14%, with 16% of men and 12% of women affected.^2^ As a chronic disease, obesity increases the risk of mortality from coronary heart disease, stroke, type 2 diabetes, chronic kidney disease, and cancers of liver, colon, kidney, endometrium, prostate, and breast.^3^ While pharmacological treatment and lifestyle modifications can promote weight loss, evidence indicates that metabolic and bariatric surgery (MBS) is currently the most durable, clinically effective, and cost‑effective treatment for all classes of obesity. MBS provides superior weight loss results as compared with nonoperative treatments, and resolves weight‑related diseases.^3^^;^^4^^;^^5^^;^^6^^;^^7^^;^^8^^;^^9^

In 2022, the American Society for Metabolic and Bariatric Surgery and the International Federation for the Surgery of Obesity and Metabolic Disorders published a joint statement redefining body mass index (BMI) thresholds for adult candidates for MBS. Currently, bariatric surgery is recommended for individuals with BMI equal to or above 35 kg/m2. It should be considered for those with BMI of 30–34.9 kg/m2 with obesity‑related comorbidities who have not responded to nonsurgical therapy, as opposed to previous criteria of BMI equal to or above 40 kg/m2 or equal to or above 35 kg/m2 with comorbidities.^3^ Former thresholds remain well‑established, because they have been standard selection criteria for MBS cost coverage in many health care systems, including Poland.^10^^;^^11^^;^^12^ Various surgical techniques have been developed, but laparoscopic sleeve gastrectomy and laparoscopic Roux‑en‑Y gastric bypass are the 2 most commonly performed procedures, accounting for approximately 80% to 90% of all MBS worldwide.^4^^;^^6^^;^^13^^;^^14^ Both approaches significantly affect weight, with expected weight loss at 2 years postsurgery of approximately 35% total body weight (up to 70% excess weight loss) and resolution of obesity‑related diseases. Sleeve gastrectomy is increas‑ ingly popular as the first‑line treatment due to similar weight loss, lower impact on nutrient absorption, and lower complication rates than Roux‑en‑Y gastric bypass.^6^^;^^15^

Adequate clinical care guided by a multidisciplinary team can enhance and maintain the long‑term effects of MBS.^16^ International guidelines suggest this optimally incorporates physical activity, dietary changes, counseling, support groups, and behavior modification techniques.^13^^;^^16^^;^^17^^;^^18^^;^^19^** **Several international societies have issued guidance documents on pre‑, peri‑, and postoperative bariatric care.^3^^;^^13^^;^^18^**^;^**^19^ Similarly, the Metabolic and Bariatric Surgery Chapter of the Association of Polish Surgeons (APS) published the Bariatric and Metabolic Surgery Care Standards in 2020, outlining expected levels of care and management for patients undergoing MBS.^11^

## AIM 

This study aimed to investigate, through a nationwide survey of Polish surgical departments performing MBS, the extent to which the Bariatric and Metabolic Surgery Care Standards are followed and to explore factors associated with better compliance.

## MATERIALS AND METHODS

###  Survey 

We designed and distributed (August–December 2022) an Internet‑based survey under the patronage of the Metabolic and Bariatric Surgery Chapter of the APS. A list of Polish surgical departments performing MBS was obtained from the official website (https://bariatria.tchp.pl/) and extended based on our knowledge of additional nonlisted centers. We contacted each center via email or phone with an invitation to participate, and posted an invitation on the official website of the Metabolic and Bariatric Surgery Chapter of the APS.

The questionnaire included comprehensive instructions and questions divided into 4 sections. Part I consisted of 10 questions concerning general characteristics of the center, including its name, contact details, reference level, types and annual number of MBS procedures performed, number of surgeons performing MBS, number of certified bariatric surgeons employed, assignment of the bariatric coordinator role, funding, and KOS‑BAR program (a program of complex specialist care over patient undergoing bariatric surgery) participation.

Part II consisted of 26 questions concerning the preoperative period, including ambulatory qualification for MBS, number of ambulatory visits, expertise of the qualifying surgeon, need for specialist consultations, availability and number of dietary and psychologist consultations, anesthesiologist consultation, ordering laboratory tests, preoperative nutritional recommendation schedule, methods for developing patient recommendations and sharing them with the patients, preoperative weight reduction, presence and accessibility of support groups, smoking and alcohol abstinence, laboratory parameters and scales concerning nutritional state and anesthesia, *Helicobacter pylori *eradication, sharing of surgical procedure details, sharing of informed consent forms and their signing, and usage of informed consent forms developed by the APS.

Part III contained 23 questions concerning the direct perioperative period, including accessibility of bariatric infrastructure, availability of bariatric equipment, postdischarge recommendations, availability and expertise of dietitians working in the unit, nutritional recommendations, a person in charge of communicating with patients, preparing and sharing general recommendation sets, physical activity recommendations, preparing and sharing postdischarge supplementation recommendation sets, timing of surgical check‑ups, referral for, number, and timing of dietary and psychological consultations, referral for, number, timing of, and possibility of telephone contact with the surgical department in emergencies.

Part IV contained 3 questions concerning the postoperative period, including the number of surgical check‑up visits, form of the visits, and laboratory tests and scales during follow‑up.

We asked that the survey is completed by a person or a group of people with the most extensive knowledge of the currently prevailing practices and standards in each center. Data were collected using an online instrument (REDCap, Nashville, Tennessee, Unites States), and the form was piloted before circulation.

### Statistical analysis

Descriptive statistics were used to summarize the characteristics of the included centers. We calculated the numbers and percentages for categorical variables. Associations between the number of general surgeons routinely performing MBS (continuous variable), the number of MBS performed annually (ordinal variable), and the hospital referral level (ordinal variable) were measured using the Spearman rank‑order correlation. We defined the strength of correlation Spearman rho (*r*s) as very strong (≥0.8), moderately strong (0.6–0.8), fair (0.3–0.5), and poor (<0.3).^20^

We identified 34 criteria that the centers should meet to comply with the Polish Bariatric and Metabolic Surgery Care Standards.^11^ We calculated each center’s compliance score, which is defined as the number of criteria fulfilled by the center divided by 34 (representing the total number of criteria).

Linear regression models with the Newey–West estimator were used to investigate the association between the compliance score and the following factors: hospital referral level, number of bariatric surgeries performed annually, number of surgeons performing MBS, funding, KOS‑BAR program participation, and availability of support groups. Due to the low number of included patients (n = 36), univariate models were created. The categories with single cases were not included in the analyses. The criterion for statistical significance was set at a *P *value below 0.05. For all statistical analyses, we used the R statistical software version

4.3.1 (R Foundation for Statistical Computing, Vienna, Austria).

### Ethical considerations

The study was conducted in accordance with the Declaration of Helsinki and its later amendments (Fortaleza). The aim of the study was outlined to the participants. The Jagiellonian University Bioethical Commission approved the study (1072.6120.128.2022).

## RESULTS

Between August and December 2022, we invited 46 surgical departments across Poland to participate in the study. If there was no response, at least 2 additional contact attempts were made. Thirty‑six centers (78.3%) completed the survey, while 2 (4.3%) reported that they no longer perform MBS, 1 (2.2%) refused participation, and 7 (15.2%) did not respond. The presented results are based on the 36 centers that completed the survey.

### General characteristics of the centers

TABLE 1 presents the characteristics of the 36 centers. On average, 3 surgeons in each center routinely performed MBS, ranging from 1 to 6 surgeons per center. The number of general surgeons performing MBS correlated positively with the number of MBS procedures performed annually (*r*s = 0.42; *P *= 0.01, indicating a fair correlation). However, there was no correlation between the number of surgeons and the hospital referral level (*r*s = –0.054; *P *= 0.79).

### Compliance score and predictors of better compliance 

**FIGURE 1:**
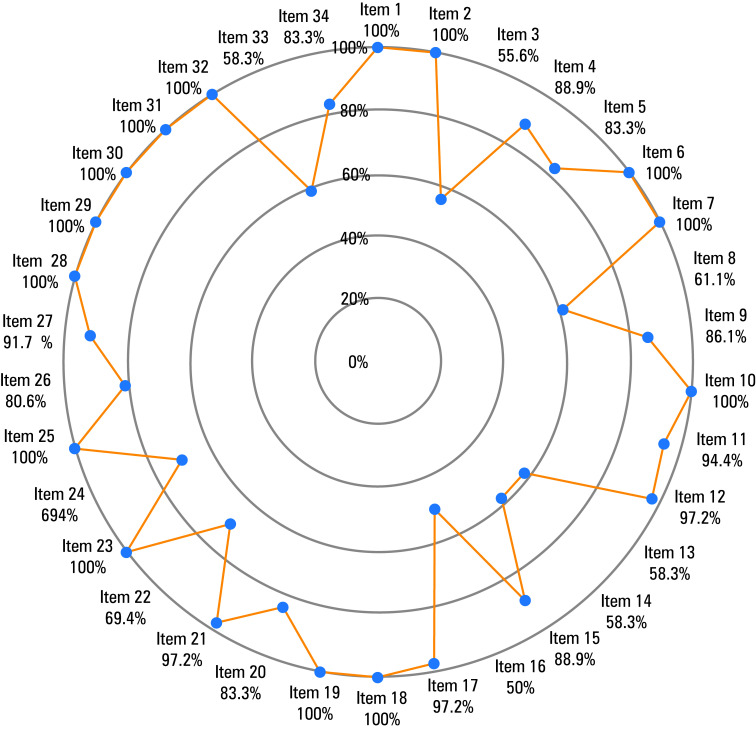
Completion of 34 items of the Bariatric and Metabolic Surgery Care Standards. Descriptions of individual items are presented in **TABLE 2**. The number below each item represents respective compliance score.

TABLE 2 lists the 34 mandatory items identified in the Bariatric and Metabolic Surgery Care Standards, and FIGURE 1 shows the completion of each item across the included centers.

Mean (SD) compliance score across the 36 centers was 86.8% (9%) (29.5/34 criteria), ranging from 70.6% to 100%. Only 3 centers (8.3%) met all 34 criteria. Sixteen centers (44.4%) met at least 90% of the criteria, and 24 (66.7%) met at least 80%. All centers adhered to 14 items, 5 items were met by 90% to 100%, 7 items were met by 80% to 90%, and 8 items were met by less than 80% of the centers.

TABLE 3  shows the factors associated with the center compliance scores. We found out that the number of surgeons performing MBS (estimated β = 2.53; 95% CI, 0.7–4.36; *P *= 0.01) and participation in the KOS‑BAR program (estimated β = 6.47; 95% CI, 0.84–12.1; *P *= 0.031) were significant predictors of better compliance.

### Pre-, peri-, and postoperative care, organizational structure, and additional considerations

The included centers demonstrated variability in several aspects of care, particularly in perioperative consultations with surgeons, dietitians, psychologists, and anesthesiologists, which are crucial for preparing patients for MBS and ensuring comprehensive care TABLE 4 . Differences were also noted in the laboratory tests, anthropometric measures, and nutritional assessment scales used for MBS qualification and postoperative check‑ups, reflecting varying clinical practices and resources TABLE 5. Preoperative instructions on nutrition and diet, essential for optimizing outcomes and ensuring adherence to recommendations, varied as well TABLE 6 . Recommendations on vitamins, supplements, diet, and physical activity provided upon discharge also differed, impacting long‑term postoperative success and nutritional health TABLE 7 . Furthermore, hospital infrastructure and equipment varied, influencing the ability to deliver state‑of‑the‑art care during the perioperative period, alongside with other unique organizational practices across the centers (TABLE 8  and TABLE 9 ).

## DISCUSSION 

Our study presents results from 36 surgical departments across Poland that perform MBS. We conducted an online survey to assess how well the Bariatric and Metabolic Surgery Care Standards, issued in 2020 by the Metabolic and Bariatric Surgery Chapter of the APS, have been implemented in practice. Mean (SD) fulfilment of the standards reached 86.8% (9%) (29.5 of 34 criteria). Individual scores ranged from 70.6% to 100%, with only 8.3% of the centers meeting all standards, and 66.7% meeting at least 80%. We identified the number of surgeons performing MBS and participation in the KOS‑BAR program as significant predictors of better compliance.

**TABLE 1:** Characteristics of the included centers

Parameter	Number of centers (%)
Hospital referral level	
I Degree (basic level hospitals that include the following departments: internal medicine, surgery, obstetrics, pediatrics, and intensive care)	8 (22.2)
II Degree (hospitals that additionally include at least 4 of the following departments: cardiology, neurology, dermatology, ophthalmology, orthopedics, urology, neurosurgery, pediatric surgery, surgical oncology)	16 (44.4)
III Degree (clinical hospitals)	11 (30.6)
Private clinics	1 (2.8)
Number of MBS procedures performed annually	
1–150	17 (47.2)
151–300	15 (41.7)
>300	4 (11.1)
Type of MBS performed	
Laparoscopic sleeve gastrectomy	35 (97.2)
Roux‑en‑Y gastric bypass	27 (75)
One anastomosis gastric bypass	16 (44.4)
Adjustable gastric band	2 (5.6)
Single anastomosis duodeno ‑ileal bypass	4 (11.1)
Single anastomosis sleeve ileal bypass	4 (11.1)
Biliopancreatic diversion by Scopinaro	0
Biliopancreatic diversion – duodenal switch	0
Gastric plication	0
Ileal interposition	0
	28 (77.8)
Funding	
Public funding only (National Health Fund)	31 (86.1)
Both public and commercial funding	4 (11.1)
Commercial funding only	1 (2.8)
Bariatric coordinator role	
Bariatric surgeon	28 (77.8)
Nonbariatric surgeon	3 (8.3)
Administrative coordinator	1 (2.8)
2 People responsible (2 bariatric surgeons or a bariatric surgeon and an administrative coordinator)	2 (5.6)
No coordinator	2 (5.6)
Participation in the publicly funded pilot KOS ‑BAR program encompassing multispecialty care over people undergoing bariatric surgery	16 (44.4)
Across I degree hospitals	2 (25)
Across II degree hospitals	7 (43.8)
Across III degree hospitals	7 (63.6)
Private clinics	0
Employing at least 1 general surgeon with a certificate of excellence issued by the Metabolic and Bariatric Surgery Chapter of the Association of Polish Surgeons^a^	18 (50)
Across I degree hospitals	3 (37.5)
Across II degree hospitals	8 (50)
Across III degree hospitals	7 (63.6)
Private clinics	0

a Median number of general surgeons with the bariatric certificate of excellence in the centers employing at least 1 surgeon with such a certificate was 1, ranging from 1 to 3.

Abbreviations: KOS ‑BAR, program of complex specialist care over patients undergoing bariatric surgery

**TABLE 2:** Mandatory items identified in the Bariatric and Metabolic Surgery Care Standards

Item	Description
Item 1	BMI ‑based assessment of eligibility for MBS
Item 2	Qualification for MBS by a surgeon performing MBS, who is a member of the bariatric team
Item 3	At least 2 (initial and final) consultations with a dietitian with experience in caring for MBS patients
Item 4	Minimum 5% preoperative reduction in the patient body weight / fat
Item 5	Presurgery visit to a psychologist with experience in caring for MBS patients including assessment of potential contraindications for surgery
Item 6	A visit to an anesthesiologist
Item 7	Performing laboratory test panel necessary for surgery under general anesthesia
Item 8	Screening for thyroid ‑stimulating hormone, cortisol, and fasting glucose levels
Item 9	Determination of glycated hemoglobin level in patients with type 2 diabetes
Item 10	Determination of nutritional status parameters used later on to evaluate the treatment results and the effects of dietary recommendations
Item 11	Scheduling of esophagogastroduodenoscopy and abdominal ultrasound
Item 12	Performing electrocardiogram
Item 13	Assessment of obstructive sleep apnea risk with the STOP ‑Bang questionnaire
Item 14	Obtaining a patient declaration of abstaining from smoking for a minimum of 6 weeks presurgery
Item 15	Obtaining patient informed consent for surgery, confirmed by signing the consent form recommended by the Association of Polish Surgeons
Item 16	A surgical bariatric team with at least 1 surgeon with a certificate of excellence issued by the Metabolic and Bariatric Surgery Chapter of the Association of Polish Surgeons
item 17	Availability of equipment adapted to patients weighing up to 250 kg: beds, an operating table, seats, armchairs, chairs, transport carts, weighing machine
Item 18	Availability of visual tracking equipment
Item 19	Availability of devices for electrosurgery – incision and hemostasis during the surgery
Item 20	Availability of anesthesia and ICU departments adapted for examining patients with obesity
Item 21	Availability of a general surgery department adapted for examining patients with obesity
Item 22	Availability of a radiology facility adapted for examining patients with obesity
Item 23	Constant access (24/7) to an emergency surgical intervention room (surgical theater) adapted to patients undergoing MBS
Item 24	Constant availability (24/7) of the emergency radiological diagnostics, including computed tomography, adapted to patients undergoing MBS
Item 25	Constant availability (24/7) of the emergency laboratory diagnostics adapted to patients undergoing MBS
Item 26	Constant availability (24/7) of the ICU adapted to patients undergoing MBS
Item 27	Constant availability (24/7) of the endoscopy unit ensuring the possibility of emergency diagnostic and therapeutic endoscopy adapted to patients undergoing MBS
Item 28	All MBS are performed a priori by laparoscopic method
Item 29	Information at discharge on the possibility of a permanent, 24/7 contact with a bariatric team representative in the postoperative period
Item 30	Information at discharge on dietary recommendations until the follow ‑up visit
Item 31	Availability of long ‑term (unlimited) specialist postoperative care within the center
Item 32	Information at discharge on the dates of at least 2 surgical follow ‑up visits (including 1 up to 30 days after surgery)
Item 33	Information at discharge on the date of at least 1 dietary consultation
Item 34	Availability of a consultation with a psychologist experienced in the management of bariatric patients in the postoperative period

Abbreviations: BMI, body mass index; ICU, intensive care unit; MBS, metabolic and bariatric surgery

We analyzed the level of compliance with each defined item to identify the areas with insufficient adherence (fulfilled by <80% of the centers). These areas included: 1) availability of a radiology facility adapted for examining patients with obesity (69.4%); 2) constant availability (24/7) of emergency radiological diagnostics, including computed tomography, for patients undergoing MBS (69.4%); 3) screening for thyroid‑stimulating hormone, cortisol, and fasting glucose levels (61.1%); 4) assessment of obstructive sleep apnea risk using the STOP‑Bang questionnaire (58.3%); 5) obtaining a patient’s declaration of abstaining from smoking for at least 6 weeks preoperatively (58.3%); 6) providing dates for at least 1 dietary consultation upon discharge (58.3%); 7) requiring at least 2 consultations (initial and final) with a dietitian experienced in bariatric care (55.6%); and 8) employing a surgeon with a bariatric certificate of excellence (50%).

**TABLE 3:** Factors associated with the center compliance score investigated with linear univariate regression models

Key factor	β	95% CI	*P *value	Adjusted R2
Hospital referral level				
I Degree (n = 8)	0	Reference	Reference	0.002
II Degree (n = 16)	–3.68	–9.86 to 2.51	0.25	
III Degree (n = 11)	0.97	–5.47 to 7.41	0.77	
Number of MBS procedures performed annually				
1–150 (n = 17)	0	Reference	Reference	0.032
151–300 (n = 15)	5.13	–0.2 to 10.46	0.07	
>300 (n = 4)	5.62	–2.56 to 13.8	0.19	
Surgeons performing MBS (n = 36)	2.53	0.7–4.36	0.01	0.11
Funding				
Both public and commercial (n= 4) vs ref. public funding only (National Health Fund) (n= 31)	1.97	–8.92 to 12.86	0.72	<0.001
KOS‑BAR program participation				
Yes (n = 16) vs ref. No (n = 20)	6.47	0.84–12.1	0.03	0.106
Availability of the support group				
Yes (n = 34) vs ref. No (n = 2)	6.31	–4.35 to 16.98	0.25	<0.001

Abbreviations: β, nonstandardized regression coefficient; others, see TABLE 2 and TABLE 3

**TABLE 4:** Aspects of surgical, dietary, psychological, and anesthesiologic consultations (continued on the next page)

Parameter	Number of centers (%)	
	Preoperatively	Postoperatively
Surgical consultation
Patient eligibility for MBS determined in		
Surgical clinic	34 (94.4)	–
Bariatric and metabolic clinic	2 (5.6)	–
Performed by		
A surgeon routinely performing MBS	36 (100)	–
Most commonly comprised		
2 visits	23 (63.9)	–
3 visits	9 (25)	–
4 visits	3 (8.3)	–
1 visit	1 (2.8)	–
Date of the first postoperative visit already set at the hospital discharge		
Yes	–	31 (86.1)
No	–	5 (13.9)
Date of the first postoperative visit scheduled		
Up to 7 days after discharge	–	10 (27.8)
Up to 8–14 days after discharge	–	21 (58.3)
Up to 15–30 days after discharge	–	4 (11.1)
Up to 30 days after discharge	–	1 (2.8)
Possibility of control visits in the form of teleconsultation		
Yes	–	29 (80.6)
No	–	7 (19.4)
Dietary consultation		
Routinely offered	36 (100)	36 (100)
Attendance		
Obligatory	29 (80.6)	21 (58.3)
Voluntary	7 (19.4)	15 (41.7)
Average number of obligatory dietary visits		
1	6 (20.7)	8 (38.1)
2	20 (69)	8 (38.1)
3 or more	3 (10.3)	5 (23.8)
Obligatory visit conducted by		
Dietician having education and experience working with patients with severe obesity	27 (93.1)	20 (95.2)
Dietician without education and experience working with patients with severe obesity	2 (6.9)	1 (4.8)
Date of the first postoperative visit already set at hospital discharge		
Yes	–	12 (57.1)
No	–	9 (42.9)
Psychological consultation		
Routinely offered	33 (91.7)	28 (77.7)
Attendance		
Obligatory	31 (93.9)	8 (28.6)
Voluntary	2 (6.1)	20 (71.4)
Average number of obligatory psychological visits		
1	18 (58.1)	3 (37.5)
2	10 (32.3)	4 (50)
3 or more	3 (9.7)	1 (12.5)
Obligatory visit conducted by		
Psychologist having education and experience working with patients with severe obesity	30 (96.8)	8 (100)
Psychologist without education and experience working with patients with severe obesity	1 (3.2)	0
Date of the first postoperative visit already set at discharge		
Yes	–	7 (87.5)
No	–	1 (12.5)
Anesthesiologist consultation		
Setting		
Hospital	19 (52.8)	–
Outpatient	12 (33.3)	–
Hospital or clinic	3 (8.3)	–
Both hospital and clinic	2 (5.6)	–

Abbreviations: see TABLE 2

**TABLE 5:** Laboratory tests, anthropometric measures, and nutritional assessment scales used for metabolic and bariatric surgery qualification and postoperative check‑ups

Parameter	Number of centers (%)	
	Preoperatively	Postoperatively
Weight assessment	31 (86.1)	33 (91.7)
BMI assessment	36 (100)	35 (97.2)
Bioelectric impedance analysis	11 (30.6)	10 (27.6)
Waist circumference measurement	13 (36.1)	12 (33.3)
Waist‑hip ratio measurement	11 (30.6)	10 (27.8)
Subjective global assessment form	9 (25)	5 (13.9)
Nutritional risk score calculation	24 (66.7)	7 (19.4)
Blood albumin level measurement	21 (58.3)	19 (52.8)
Total blood protein measurement	21 (58.3)	20 (55.6)
Schedule of obligatory preoperative laboratory tests		
Clinic	21 (58.3)	–
Hospital	12 (33.3)	–
Anesthesiologist clinic	1 (2.8)	–
Clinic screening with repetition in the hospital	2 (5.6)	–

Abbreviations: see TABLE 2

Severe obesity is a chronic disease requiring adequate long‑term care guided by a multidisciplinary team. Studies have demonstrated that multidisciplinary care is essential to standardizing management and reducing complication rates.^18^^;^^21^^;^^22^ Including a registered dietitian and a licensed psychologist with expertise in the bariatric care is crucial for adequately preparing patients for MBS and ensuring appropriate pre‑, peri‑, and postoperative support.^3^^;^^18^^;^^23^ Our results indicate that Polish surgical departments highly value dietary and psychological support. All patients were routinely offered dietary consultations pre‑ and postoperatively. The preoperative dietary consultation was mandatory in 80.6% of the centers, and these visits were conducted by a dietitian with experience in patients with severe obesity in 93.1% of the cases. The postoperative dietary consultation was mandatory in 58.3% of the centers, with 95.2% ensuring consultation with a trained dietitian.

**TABLE 6:** Aspects of preoperative instructions on nutrition and diet

Parameter	Number of centers (%)
Information regarding required nutrition and diet is provided in	
Surgical clinic	15 (41.7)
Surgical clinic and dietary clinic	13 (33.3)
Dietary clinic	8 (22.2)
Prehabilitation clinic	1 (2.8)
Centers that provide instructions on nutrition and diet within a surgical clinic (n = 27; 75%) Form of instruction	
Cohesive set of instructions	26 (96.3)
No particular scheme	1 (3.7)
Medium of instruction	
Printed	22 (81.5)
Oral	3 (11.1)
Link to a website with instructions	1 (3.7)
Bariatric book	1 (3.7)
Person developing the instructions	
Both physicians and dieticians	20 (74.1)
Physicians alone	2 (11.1)
Dieticians alone	3 (11.1)
Multispecialty bariatric team	

**TABLE 7:** Recommendations on vitamins, supplements, diet, and physical activity provided upon discharge

Parameter	Number of centers (%)
Recommendations regarding vitamins, supplements, and diet	36 (100)
Delivered by	
Treating physician	14 (38.9)
Clinical dietician	4 (11.1)
Clinical dietician dedicated to patients undergoing bariatric surgery	16 (44.4)
Bariatric surgeon	1 (2.8)
Nurse	1 (2.8)
Medium of instructions	
Printed	35 (97.2)
Link to a website	1 (2.8)
Person developing the instructions	
Both physicians and dieticians	26 (72.2)
Physicians alone	4 (11.1)
Dieticians alone	5 (13.9)
Multispecialty bariatric team	1 (2.8)
Recommendations regarding physical activity	34 (94.4)
Medium of instructions	
Printed	17 (52.9)
Oral	15 (44.1)
Not delivered	1 (2.9)
Person developing the instructions	
Both physicians and physiotherapists	18 (52.9)
Physicians alone	9 (26.5)
Physiotherapists alone	4 (11.8)
Bariatric team alone	1 (2.9)
No formalized scheme was developed	2 (5.9)

**TABLE 8:** Hospital infrastructure and equipment across the included centers

Parameter		Number of centers (%)
Adequate equipment to manage bariatric patients in		
Intensive care unit		30 (83.3)
Surgical ward (eg, transporting equipment with load capacity up to 250 kg)		35 (97.2)
Radiological ward equipped adequately to manage bariatric patients		25 (69.4)
Laparoscopic bariatric surgery instrument set		36 (100)
24‑hour access to		
Surgical ward at the same hospital		36 (100)
Intensive care unit at the same hospital		34 (94.4)
Computed tomography	With a radiologist on ‑site	25 (69.4)
	With a radiologist on‑call	4 (11.1)
	With teleradiology	11 (30.6)
	Outside the unit	3 (8.3)
Surgical theater		36 (100)
Analytical laboratory		36 (100)
Endoscopy unit	With an endoscopist on‑site	15 (41.7)
	With an endoscopist on‑call	18 (50)

Additionally, 91.7% and 77.7% of the centers routinely offered psychological consultations pre‑ and postoperatively, respectively. The preoperative consultation was mandatory in 93.9% of the centers, with 96.8% ensuring a consultation with an experienced psychologist. The postoperative consultation was mandatory in 28.6% of the centers, with all visits conducted by a trained psychologist. These findings indicate that ensuring multidisciplinary perioperative care is considered important within Polish surgical departments; however, there is a need to increase the availability of dietary and psychological consultations in the postoperative period, as these are crucial for patient recovery and long‑term success.

Guidelines indicate that patients undergoing MBS should receive nutritional and physical activity specialist care starting in the early postoperative period.^18^^;^^19^ The centers included in this study complied well with these standards, as all of them provided recommendations regarding vitamins, supplements, and diet upon discharge, and 94.4% provided recommendations on physical activity.

The introduction of standards and recommendations reflects positively on the quality of care and results. Implementation of the Metabolic and Bariatric Surgery Accreditation and Quality Improvement Program in the United States and Canada in 2016, was associated with significant reductions in the rates of 30‑day surgical and medical complications and greater likelihood of shorter hospital stays.^24^ Similarly, introduction of enhanced recovery program for bariatric surgery patients resulted in a decrease in the length of hospital stay, cost, and risk of 30‑day readmission.^25^^;^^26^^;^^27^ Introduction of standards of care and clinical protocols results in improving health care and outlines goals for hospitals and clinical centers to pursue.

A major strength of our study is the inclusion of most Polish surgical departments performing MBS, as we collected data from 36 (78.3%) of 46 identified centers. This allowed us to gain insight into current practices, and to identify similarities and differences in the organization of pre‑, peri‑, and postoperative care, which may be useful for decision makers. We also identified areas that need improvement.

Our study has some limitations. We requested that the survey is completed by the person or group with the best insight into the prevailing standards and practices at each center, which may not represent the full range of practices at all levels of care. Additionally, the data reported by the center representatives might not be objectively verified and may include estimated values, as we collected information via survey without requesting documentation. Finally, the results pertain exclusively to Polish surgical departments performing bariatric surgery and should not be generalized beyond the Polish health care system.

## CONCLUSIONS 

Our study indicates that the Bariatric and Metabolic Surgery Care Standards are moderately well adopted by Polish surgical departments performing bariatric surgery, with an average completion of 86.8% of all 34 items. The number of surgeons performing MBS and participation in the KOS‑BAR program were significant predictors of better compliance with the standards. We identified 8 areas that were inadequately addressed by less than 80% of the centers, indicating room for improvement. Unifying prevailing standards across Polish surgical departments and adopting existing Polish and international guidelines more broadly may lead to better patient treatment outcomes.

**TABLE 9:** Additional organizational practices and aspects of perioperative care

Parameter	Number of centers (%)
Requirement of preoperative weight reduction	
At least 5%	32 (88.9)
At least 3%–5%	1 (2.8)
To lose as much weight as possible, disqualification only when a patient gains weight	2 (5.6)
To keep the weight stable	1 (2.8)
Obligatory abstaining from smoking before surgery	30 (83.3)a
Obligatory for at least 6 weeks	21 (58.3)
Obligatory abstaining from alcohol before surgery	20 (55.6)b
Obligatory for at least 6 weeks	15 (41.7)
Routine eradication in the case of *Helicobacter pylori *detection in preoperative gastroscopy	
Obligatory	27 (75)
Suggested but not obligatory	9 (25)
Information regarding the course of surgical procedure, perioperative risks, and potential complications outlined to patients	
In the surgical clinic	34 (94.4)
In the hospital	2 (5.6)
Informed consent form available to patients to familiarize with	
At home	19 (55.6)
In the hospital	16 (44.4)
Incorporation of consent forms endorsed by the Association of Polish Surgeons’ website	
Yes	32 (88.9)
No	4 (11.1)
Dedicated support group for patients undergoing MBS	
Available within the clinic	20 (55.6)
A local support society unrelated to the clinic / hospital	15 (38.9)
No group available	2 (5.6)
Informing patients on the possibility of permanent, 24/7 phone contact with the bariatric team after discharge	36 (100)
Patients are provided with a phone number to a member of the bariatric team	23 (63.9)
Patients are provided with a phone number to the doctor’s office in the surgical ward	13 (36.1)

a Range of minimum period abstaining from smoking was between 2 and 12 weeks.

b Range of minimum period abstaining from alcohol was between 2 and 12 months.
